# Glances at the Hospitals

**Published:** 1904-09-17

**Authors:** 


### GLANCES AT THE HOSPITALS.

### THE LEICESTER INFIRMARY.

WHEN our "Glance" at the Leicester Infirmary appeared we had not received the medical and surgical statistics which at this institution are printed in a separate report. This has now reached us and we are pleased to notice it as it is one of the most carefully-prepared and clearly-arranged set of tables we have yet met with. There are separate columns showing the sex, age, and results of treatment in every group of diseases, and at the end of the report there are 34 pages of notes giving details of 40 of the most interesting medical and surgical cases of the year. It is much to be desired that every provincial infirmary of, say, over 100 beds, would follow the example of the Leicester Infirmary, and present us with equally useful statistics. As an instance of their accuracy we notice that removal of ovarian cyst was performed 23 times, and the columns show that 1 of these cases was under twenty years of age; 6 were under thirty; 7 under forty; 2 under fifty; 6 under sixty, and 1 over sixty. Of the total 20 were cured, 2 died, and 1 remained under treatment. Again, appendectomy was performed 23 times. Of these 22 were successful and death followed in one case. The operation for the radical cure of hernia was performed 92 times and all were successful. In the medical wards 64 cases of acute rheumatism were under treatment. Of these 6 were under twelve years of age; 5 under fifteen years; 14 under twenty; 26 under thirty; 5 under forty; 6 under fifty, and 2 were about sixty. Among this group 55 are returned as cured, and 9 as being under treatment at the date of the report. Rheumatic fever is a very important disease for every medical practitioner, and at the risk of being thought a trifle hypercritical or too inquisitive, we should like to have had a note to this table stating the treatment pursued; the duration in the hospital, and the percentage having heart complications. From the anæsthetic table we learn that chloroform was the agent used in 1,262 instances; ether in 978; gas in 361; ether and chloroform in 75; chloroform and A.C.E. in 20; A.C.E. in 12, and gas and ether in 6. The total number of operations performed on in-patients was 1,599.

CHELTENHAM GENERAL HOSPITAL.

THE total receipts amounted to £5,772, and the expenditure exceeded this by £1,889. The annual subscriptions were £1,819, the donations £423, collections in churches and chapels £607, and Saturday Fund, etc., £160; but it does not appear what sum the workpeople themselves contributed to the subscription list. In some hospitals this sum is a very substantial one. The total number of in-patients under treatment during the year was 771. Of this number 364 were discharged cured, 101 were discharged relieved, 27 for various reasons, 57 died, and 59 remained in the wards at the end of the year. The average cost of each in-patient was £6 3s. 3d., and of each out-patient 4s. 7d. The average daily number resident in the hospital was 47, and the average stay in the wards was 22 days. The medical and surgical tables are very good, the results are given, and there is an additional and useful column for remarks. There were 14 cases of gastric ulcer, of which 11 were cured and 3 remained under treatment. Of 33 cases of pneumonia or broncho-pneumonia 24 were cured, 1 relieved, 4 died, and 4 remained in the wards. Of 4 cases of ovariotomy 2 were cured, 1 relieved, and 1 died. There is no anæsthetic table.

